# Supplementary material for: Curcumin-loaded nanocomplexes ameliorate the severity of nonalcoholic steatohepatitis in hamsters infected with Opisthorchis viverrini
Source: PLoS One. 2022 Sep 27;17(9):e0275273. doi: 10.1371/journal.pone.0275273 (PMC9514634; doi:10.1371/journal.pone.0275273)
Supplement: S1 Table — (DOCX) [file pone.0275273.s001.docx]

**Supporting information**

**Table S1.** Nutrient composition of HFF diet.

| List test | Results of test | Units |
| --- | --- | --- |
| Total carbohydrate | 56.12 | g/100g |
| Total sugar | 39 | g/100g |
| Total fat | 21.5 | g/100g |
| Cholesterol | 978.85 | mg/100g |
| Protein | 10.58 | g/100g |
| Moisture | 9.27 | g/100g |
| Crude fiber | 0.63 | g/100g |
| Energy | 460.30 | Kcal/100g |
