# Supplementary figures and images for: Curcumin-loaded nanocomplexes ameliorate the severity of nonalcoholic steatohepatitis in hamsters infected with Opisthorchis viverrini
Source: PLoS One. 2022 Sep 27;17(9):e0275273. doi: 10.1371/journal.pone.0275273 (PMC9514634; doi:10.1371/journal.pone.0275273)

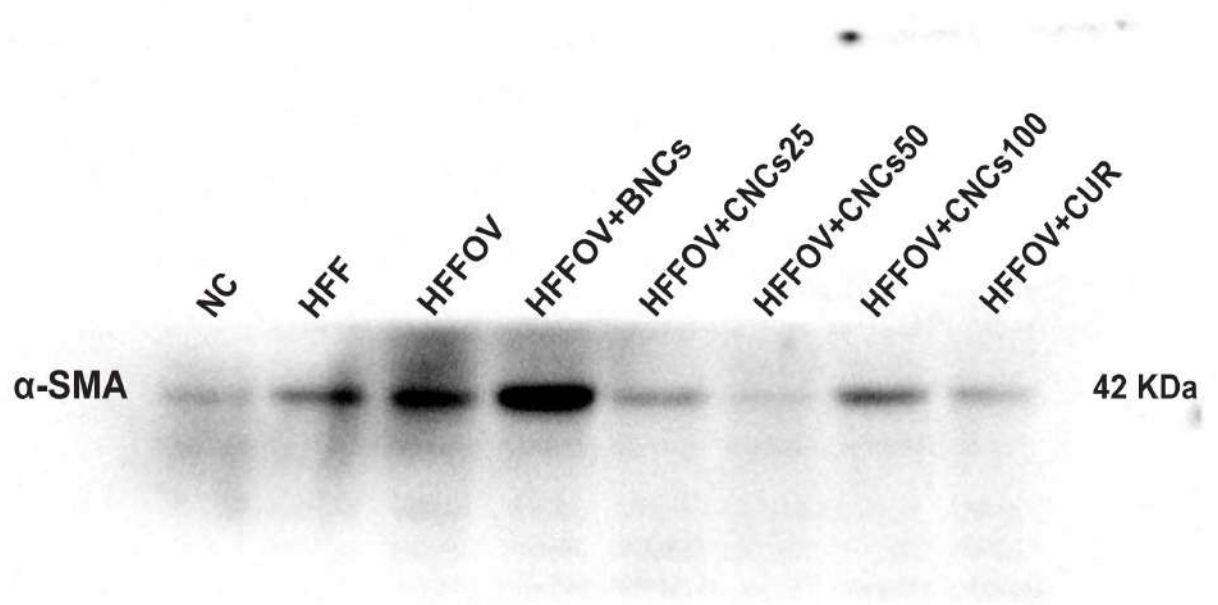

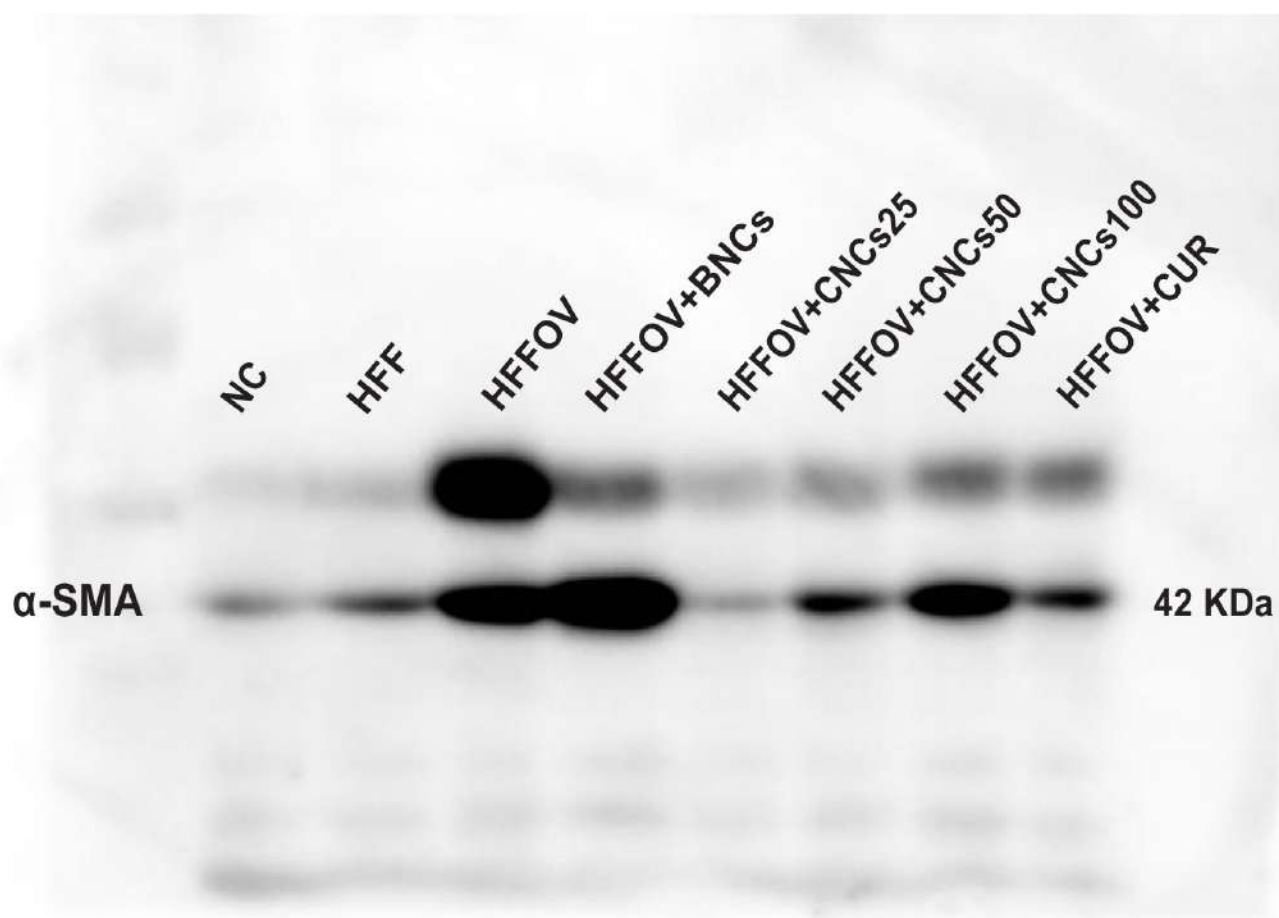

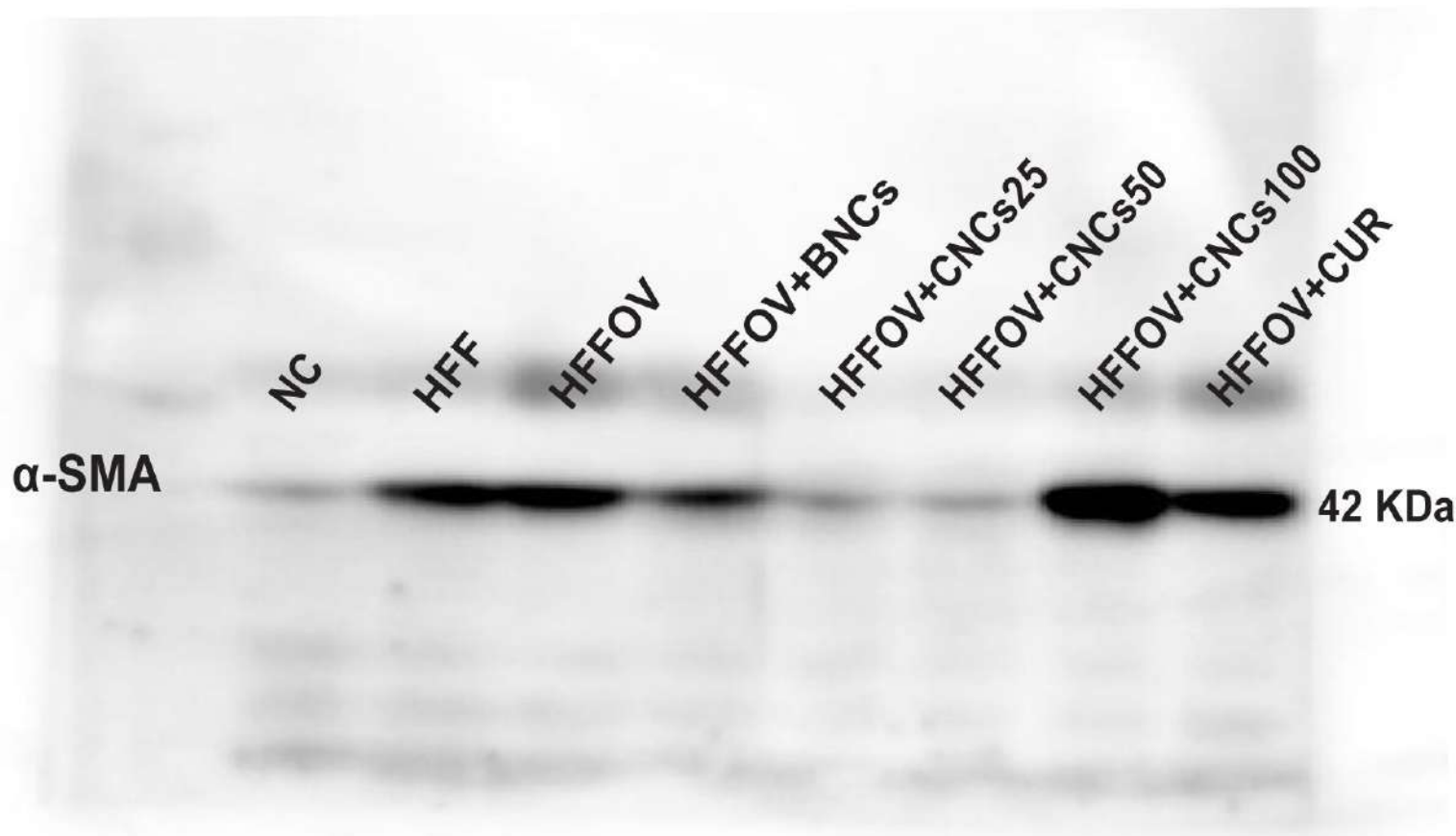

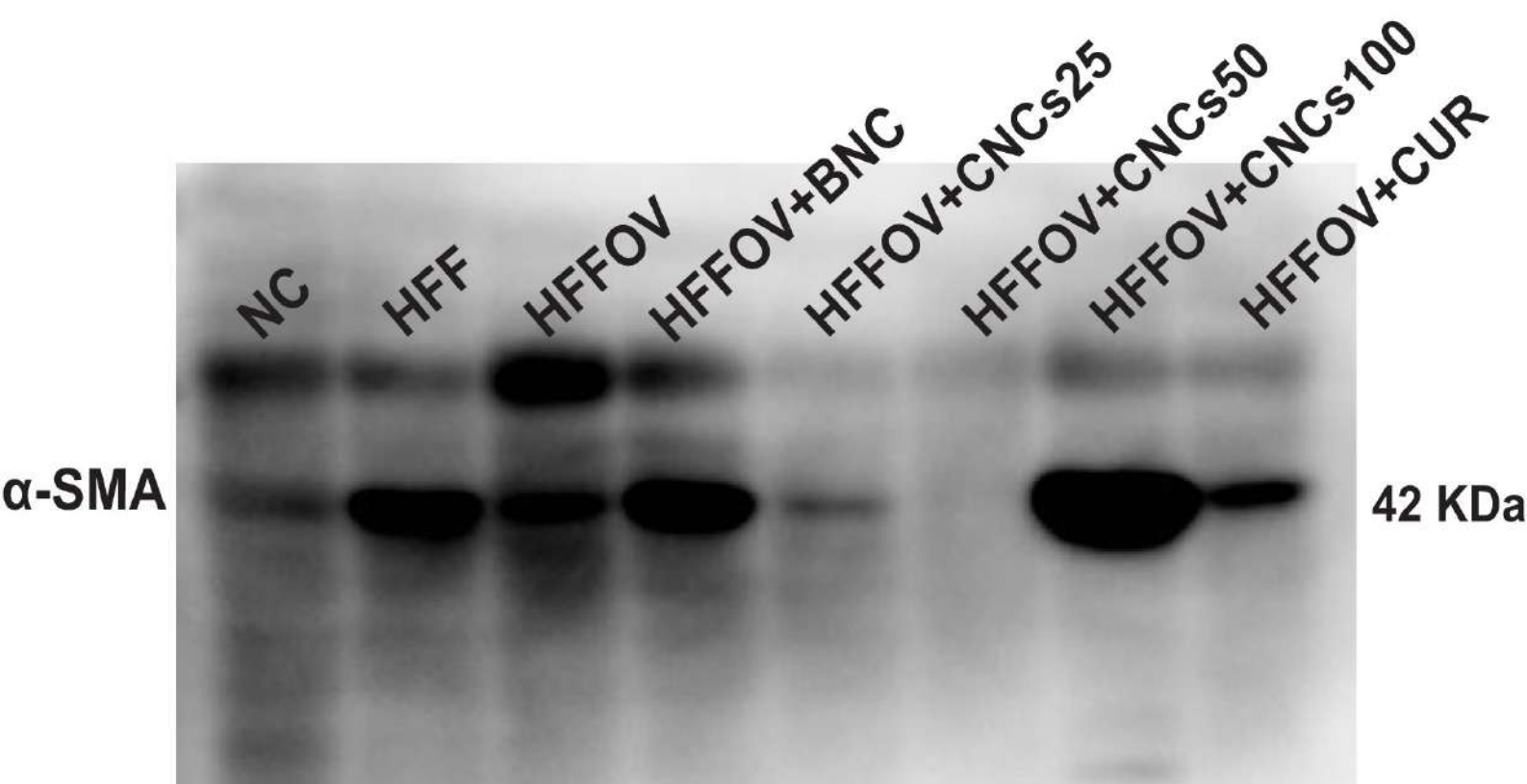

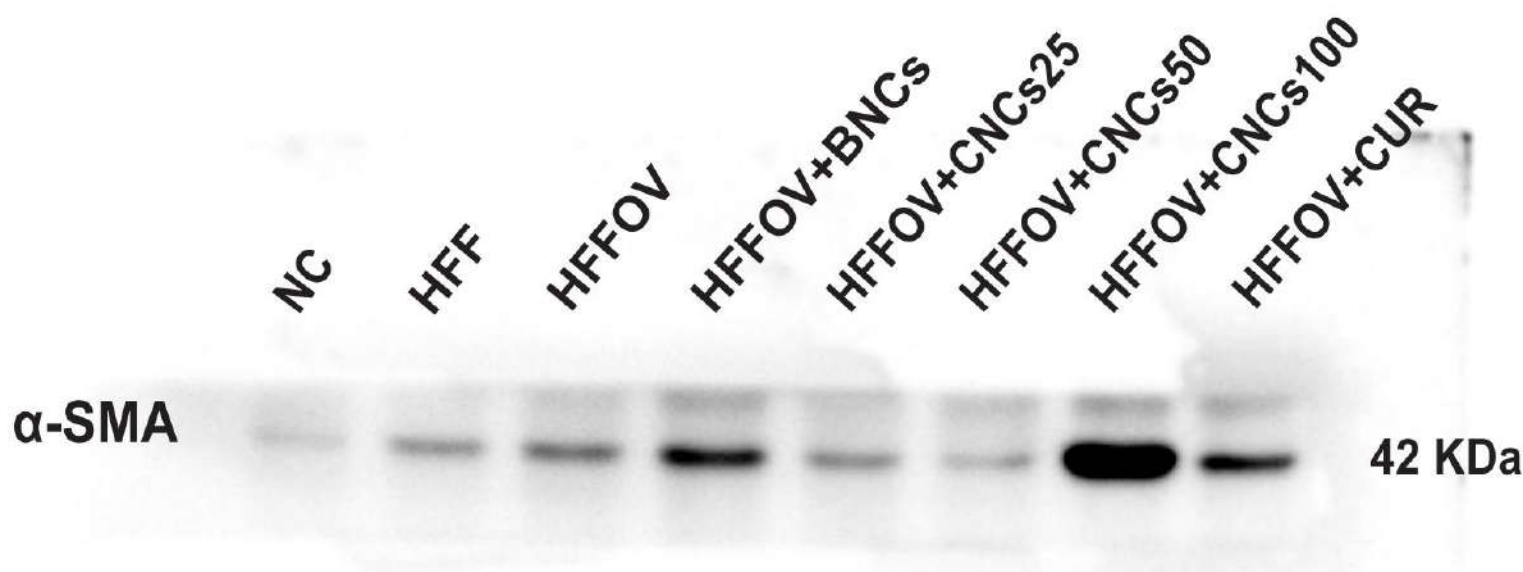

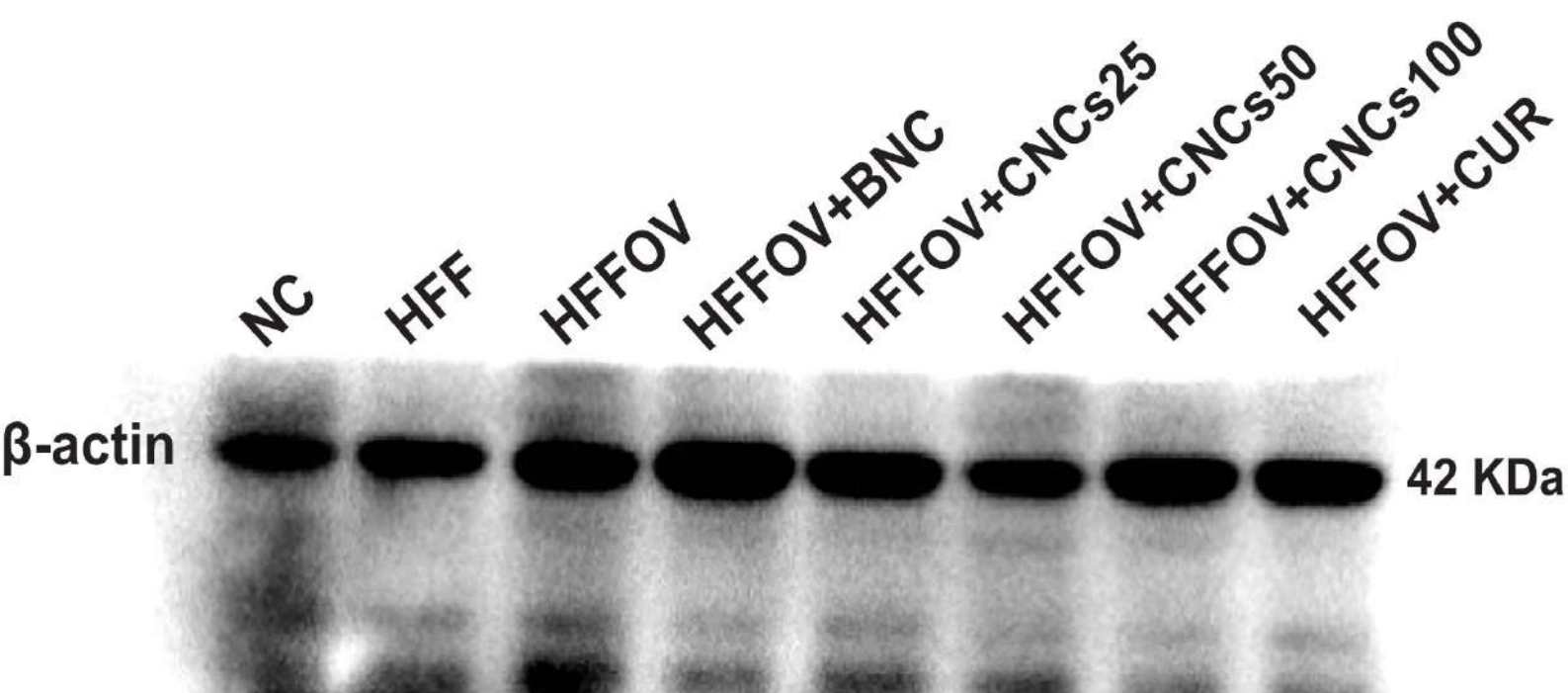

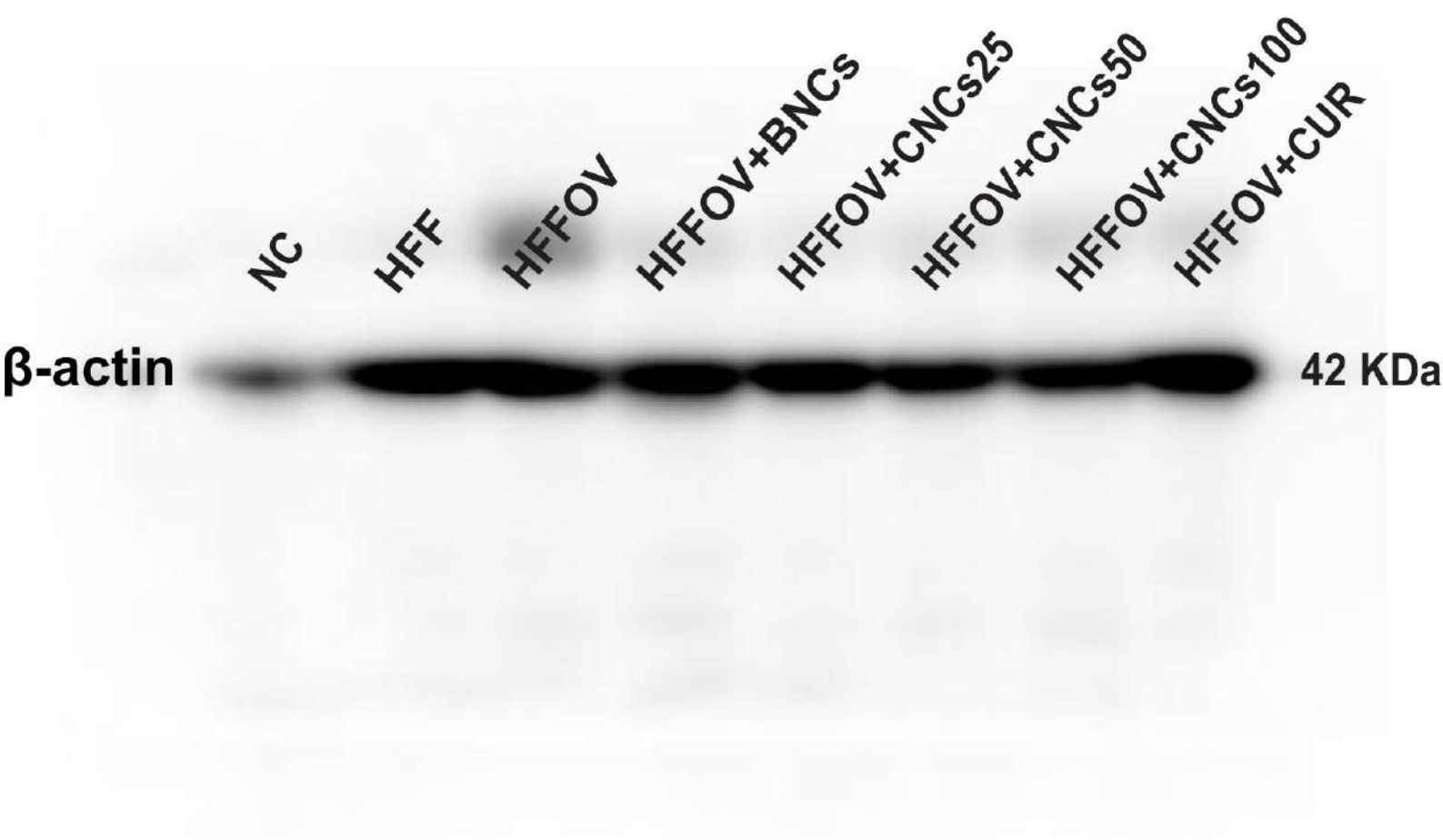

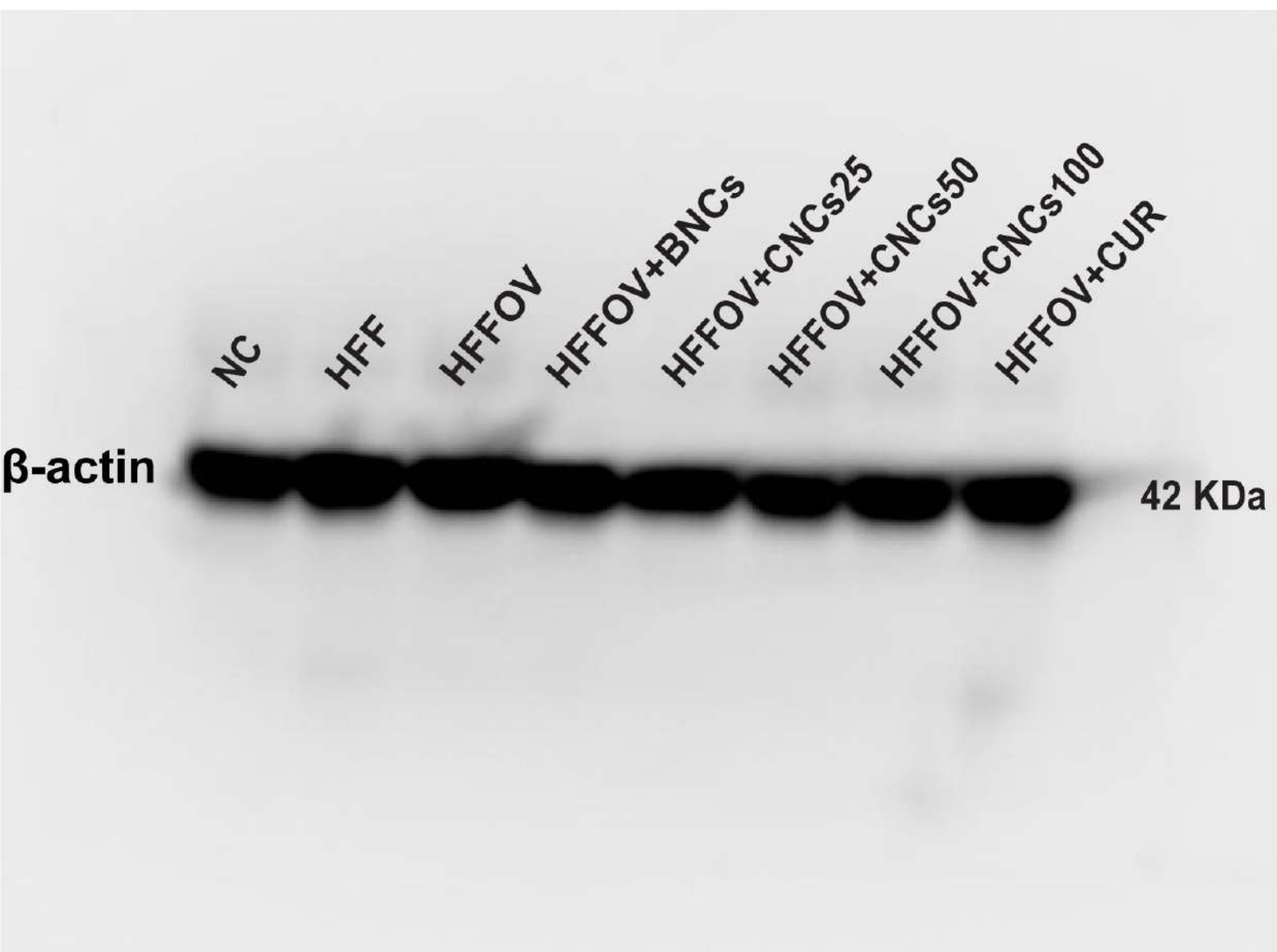

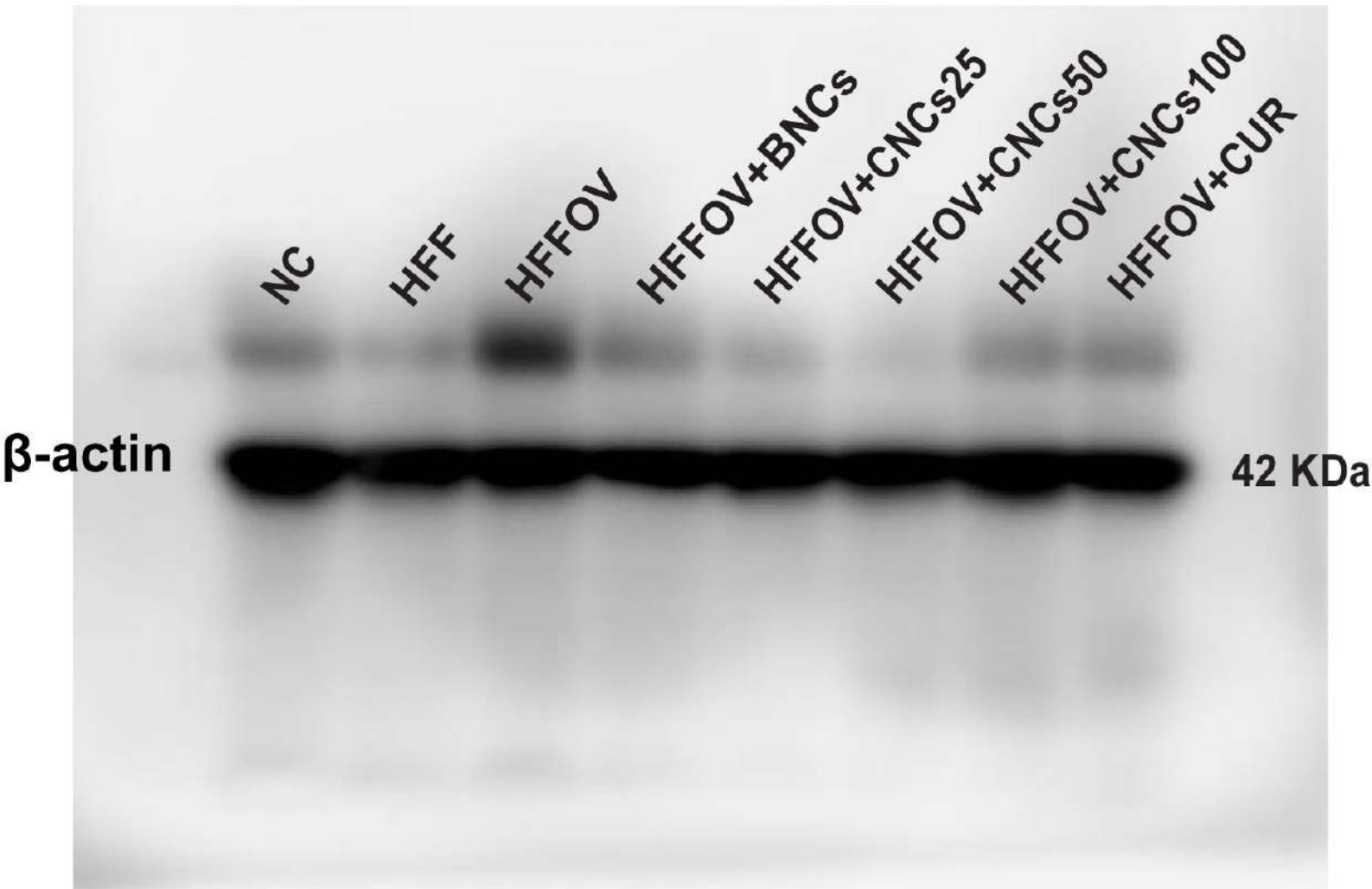

**β-actin**

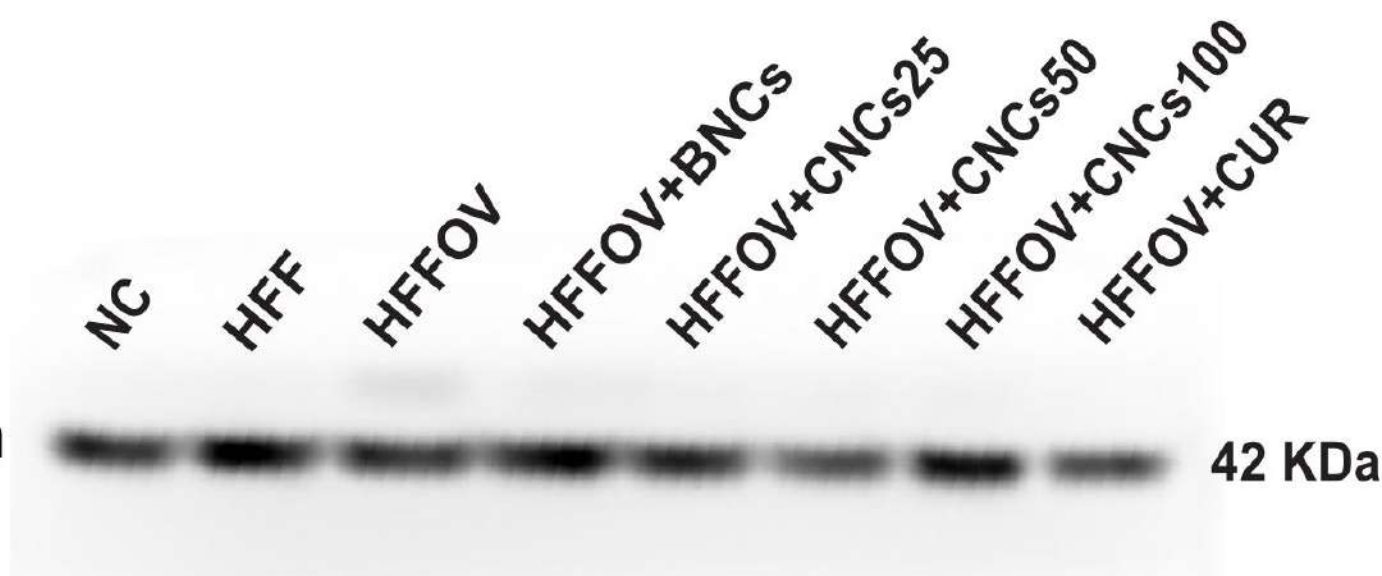

Supplement: S1 Fig — (PDF) [file pone.0275273.s002.pdf]
