# Supplementary material for: Curcumin-loaded nanocomplexes ameliorate the severity of nonalcoholic steatohepatitis in hamsters infected with Opisthorchis viverrini
Source: PLoS One. 2022 Sep 27;17(9):e0275273. doi: 10.1371/journal.pone.0275273 (PMC9514634; doi:10.1371/journal.pone.0275273)
Supplement: Attachment — Submitted filename: Response to reviewer-PONE-D-22-11833R1.docx [file pone.0275273.s003.docx]

PONE-D-22-11833

Curcumin-loaded nanocomplexes ameliorate the progression of NAFLD to NASH in hamsters infected with *Opisthorchis viverrini*

Title has been changed to “Curcumin-loaded nanocomplexes ameliorate the severity of nonalcoholic steatohepatitis in hamsters infected with *Opisthorchis viverrini*”

Response to the Journal Requirements:

When submitting your revision, we need you to address these additional requirements.

1. Please ensure that your manuscript meets PLOS ONE's style requirements, including those for file naming. The PLOS ONE style templates can be found at

<https://journals.plos.org/plosone/s/file?id=wjVg/PLOSOne_formatting_sample_main_body.pdf>  and

<https://journals.plos.org/plosone/s/file?id=ba62/PLOSOne_formatting_sample_title_authors_affiliations.pdf>

Response: We have rechecked and ensured that the manuscript meets PLOS ONE’s style requirements, including those for file naming.

2. In your Data Availability statement, you have not specified where the minimal data set underlying the results described in your manuscript can be found. PLOS defines a study's minimal data set as the underlying data used to reach the conclusions drawn in the manuscript and any additional data required to replicate the reported study findings in their entirety. All PLOS journals require that the minimal data set be made fully available. For more information about our data policy, please see <http://journals.plos.org/plosone/s/data-availability>.

"Upon re-submitting your revised manuscript, please upload your study’s minimal underlying data set as either Supporting Information files or to a stable, public repository and include the relevant URLs, DOIs, or accession numbers within your revised cover letter. For a list of acceptable repositories, please see <http://journals.plos.org/plosone/s/data-availability#loc-recommended-repositories>. Any potentially identifying patient information must be fully anonymized.

Important: If there are ethical or legal restrictions to sharing your data publicly, please explain these restrictions in detail. Please see our guidelines for more information on what we consider unacceptable restrictions to publicly sharing data: <http://journals.plos.org/plosone/s/data-availability#loc-unacceptable-data-access-restrictions>. Note that it is not acceptable for the authors to be the sole named individuals responsible for ensuring data access.

We will update your Data Availability statement to reflect the information you provide in your cover letter.

Response: Our Data Availability statement can now be found as supportive information.

3. Thank you for stating the following financial disclosure:

“This study was supported by the Research and Researchers for Industries (RRI: MSD62I0041), Thailand Science Research and Innovation (TSRI: RDG6250045) and Mekong Health Science Research Institute Khon Kaen University (MeHSRI09/2561).”

Please state what role the funders took in the study.  If the funders had no role, please state: "The funders had no role in study design, data collection and analysis, decision to publish, or preparation of the manuscript."

If this statement is not correct you must amend it as needed.

Please include this amended Role of Funder statement in your cover letter; we will change the online submission form on your behalf.

Response: we have stated the role of funders as suggested: “The funders had no role in study design, data collection and analysis, decision to publish, or preparation of the manuscript.”

4. PLOS ONE now requires that authors provide the original uncropped and unadjusted images underlying all blot or gel results reported in a submission’s figures or Supporting Information files. This policy and the journal’s other requirements for blot/gel reporting and figure preparation are described in detail at <https://journals.plos.org/plosone/s/figures#loc-blot-and-gel-reporting-requirements> and <https://journals.plos.org/plosone/s/figures#loc-preparing-figures-from-image-files>. When you submit your revised manuscript, please ensure that your figures adhere fully to these guidelines and provide the original underlying images for all blot or gel data reported in your submission. See the following link for instructions on providing the original image data: <https://journals.plos.org/plosone/s/figures#loc-original-images-for-blots-and-gels>.

In your cover letter, please note whether your blot/gel image data are in Supporting Information or posted at a public data repository, provide the repository URL if relevant, and provide specific details as to which raw blot/gel images, if any, are not available. Email us at [plosone@plos.org](mailto:plosone@plos.org) if you have any questions.

Response: We have provided our raw blot/gel image data in Supporting information as is required.

5. Please review your reference list to ensure that it is complete and correct. If you have cited papers that have been retracted, please include the rationale for doing so in the manuscript text, or remove these references and replace them with relevant current references. Any changes to the reference list should be mentioned in the rebuttal letter that accompanies your revised manuscript. If you need to cite a retracted article, indicate the article’s retracted status in the References list and also include a citation and full reference for the retraction notice.

Response: we have re-checked all references and all are correct.

**Response to the Reviewers' comments:**

Reviewer #1: The authors investigated the effect of nanoencapsulated and natural curcumin on liver injuries and the development of nonalcoholic fatty-liver disease in combination with OV liver fluke infection and a high-fat high-fructose diet. The strength of the study is the number of groups, including three different doses of nanoencapsulated curcumin and the number of animals within the groups (7 individuals). In general, the authors show the results of the original research and the data obtained may be of interest to the researchers in similar areas. Nevertheless, I believe that the text of the manuscript needs a revision.

Response: We thank the reviewer for their critical evaluation and suggestions for improving the manuscript. We would like to respond to each point as follows.

1. Fig.5 - there is not any indications on diagrams whether something is changing significantly or not. Accordingly, only those results that are significant should be described in the text, so (lines 272-281) ...” The expression level of CD36 was high in the HFFOV group compared with the HFF group (Fig 5A) and was lower than this in all groups receiving treatment (with BNCs, CNCs or CUR).” Are there statistically significant differences between each of these groups to draw such conclusions?

Response: We have reanalyzed and revised Fig 5A (old version, now Fig 7A in the new version). Text was also revised in line with this change.

2. Similar question applies to Fig4C - is there any significant difference among the groups? If there are no statistical changes, **then there should also be no reason to indicate in the text that one group is higher or lower.** At present, in the results, discussion, and even in the abstract, **some text states about non-significant changes between groups.** Authors should carefully revise the text of the manuscript and remove all such statements about statistically insignificant changes. Moreover, where there is no significant difference, it could be written that **there were no significant differences among groups.** Otherwise, it will be a misinterpretation of the obtained results.

Response: We have reanalyzed and revised Fig 4 (old version, now Fig 6 in the new version). Text was also revised in line with this change.

3. Table 2 can be transferred to the supplements

Response: We have moved Table 2 to the supplements where it is referred to as Table S1.

4. It is unclear, what criteria the authors used to clearly distinguish NAFLD and NASH, could the author provide any criteria in Table 3 how it is possible to separate these two pathologies using the hamster model? It would be very helpful if the authors modified the Table 3 and inserted NAFLD and NASH criteria in the histological scoring system.

Response: We have revised Table 3 (old version, now Table 2 in the new version) and inserted the criteria used to distinguish between NAFLD and NASH as suggested.

5. There are many abbreviations in the abstract, while some of them are given without decoding, for example, HFF diet (HFF); TC and TG.

Response: We have amended the abstract accordingly in the revised version.

6. The company name and catalogue code of the primary and secondary antibodies used should be indicated in the M&M.

Response: We have added this information in the revised version.
